# Total Cellular ATP Production Changes With Primary Substrate in MCF7 Breast Cancer Cells

**DOI:** 10.3389/fonc.2020.01703

**Published:** 2020-11-02

**Authors:** Maggie C. Louie, Justin Ton, Maurice L. Brady, Diem T. Le, Jordon N. Mar, Chad A. Lerner, Akos A. Gerencser, Shona A. Mookerjee

**Affiliations:** ^1^Department of Natural Sciences and Mathematics, Dominican University of California, San Rafael, California, CA, United States; ^2^Department of Biological and Pharmaceutical Sciences, Touro University California College of Pharmacy, Vallejo, CA, United States; ^3^Buck Institute for Research on Aging, Novato, CA, United States

**Keywords:** glycolysis, oxidative phosphorylation, Crabtree, ATP supply flexibility, bioenergetic capacity

## Abstract

Cancer growth is predicted to require substantial rates of substrate catabolism and ATP turnover to drive unrestricted biosynthesis and cell growth. While substrate limitation can dramatically alter cell behavior, the effects of substrate limitation on total cellular ATP production rate is poorly understood. Here, we show that MCF7 breast cancer cells, given different combinations of the common cell culture substrates glucose, glutamine, and pyruvate, display ATP production rates 1.6-fold higher than when cells are limited to each individual substrate. This increase occurred mainly through faster oxidative ATP production, with little to no increase in glycolytic ATP production. In comparison, non-transformed C2C12 myoblast cells show no change in ATP production rate when substrates are limited. In MCF7 cells, glutamine allows unexpected access to oxidative capacity that pyruvate, also a strictly oxidized substrate, does not. Pyruvate, when added with other exogenous substrates, increases substrate-driven oxidative ATP production, by increasing both ATP supply and demand. Overall, we find that MCF7 cells are highly flexible with respect to maintaining total cellular ATP production under different substrate-limited conditions, over an acute (within minutes) timeframe that is unlikely to result from more protracted (hours or more) transcription-driven changes to metabolic enzyme expression. The near-identical ATP production rates maintained by MCF7 and C2C12 cells given single substrates reveal a potential difficulty in using substrate limitation to selectively starve cancer cells of ATP. In contrast, the higher ATP production rate conferred by mixed substrates in MCF7 cells remains a potentially exploitable difference.

## Introduction

The ability of cancer cells to respond and adapt to available substrate conditions is widely studied (1–3), with the primary goals of using cancer cell metabolism to develop diagnostic and therapeutic strategies. Like any cell, cancer cells must respond to acute fluctuations in substrate availability to meet the demands for energy and intermediates required to survive and proliferate (4–6). Over longer time frames, cancer cells adapt to longer-term effects of the microenvironment with extensive transcriptional and architectural remodeling that enables unrestricted growth in different tissues and under wide ranges of conditions. A better understanding of both the acute metabolic responses and longer-term metabolic remodeling is critical to identifying and selectively targeting metabolism in cancer cells (7, 8).

Though targeting the metabolic alteration that occurs in cancer is a long-pursued goal [recently reviewed in (9, 10)], a successful therapeutic strategy based on metabolic disruption remains unrealized. Though many observations suggest that “cancer cell metabolism” is sufficiently different from normal cellular metabolism to be selectively targetable, the wide metabolic variation among cancers in different tissues, and even cells within a tumor, makes it difficult to test this hypothesis (11–14). Differences that are described as characteristic of cancer are difficult to interpret. The Warburg effect, for example, characterized by high rates of glycolysis and/or glucose uptake (15), has persisted for almost a century (16) despite its ambiguous definition and non-testable construction with respect to cellular bioenergetic behavior (17). Proposed mechanisms for the Warburg effect include altered expression of proteins, including levels or isoforms of glycolytic enzymes (18), the mitochondrial pyruvate carrier (19) as a wide variety of specific transcriptional alterations (20). Taken together, no cohesive model emerges. Moreover, the hallmark Warburg effect characteristic of high rates of glucose flux is ambiguous: while faster glycolysis could represent a consistent shift toward glycolysis, it could also represent higher ATP demand with no alteration of catabolic machinery.

An additional problem is the highly responsive nature of metabolic networks. Unlike earlier models of cancer metabolism that proposed an irreversible “switch,” most recent models recognize the high degree of metabolic flexibility that allows a cancer cell to meet energy and biosynthesis demands even under rapidly changing conditions (21, 22). Whether this is more true in cancer cells than other cells is not clear, but limiting such flexibility should be an advantage if it can be made selective to cancer. Here, the homeostatic control over steady-state metabolic flux is a necessary consideration.

Metabolic rates (e.g., rate of glucose catabolism) are controlled by the concentrations of common intermediates in linked reactions (e.g., glycolysis) and activities of reactions linking these intermediates, which are influenced (23) but cannot be predicted (24) solely by the isoforms and abundance of the enzymes that carry out those reactions (e.g., hexokinase), as both concentrations and activities influence these rates. Analysis of proteomic and transcriptomic data provide a good understanding of the changes in metabolic machinery that occur in cancer cells (25), but not of the kinetic behavior of the metabolic system (26). Distinguishing between the plastic remodeling of metabolic networks via protein expression changes and the kinetic function of the network would greatly strengthen the hypothesis-testing ability of both conceptual and mathematical metabolic models.

The energy needs of cancer cells are a specific topic of interest within the larger field of cancer metabolism, where ATP turnover is presumably increased to meet increased the energy demand of rapidly growing and dividing cells. Although faster ATP turnover has been inferred from high rates of glycolysis in many cancer models [e.g., (27)], clear demonstration of this is currently lacking. One reason is that many assessments of energy supply and demand in whole cells end at the raw rates of associated fluxes, such as oxygen consumption and extracellular acidification. Because these rates are differently geared to ATP, and because the units (e.g., pH to moles O_2_) are not directly comparable, no combination of these raw rates can yield useful information about total cellular ATP production when both pathways are operating. In addition, each of these rates is confounded by non-ATP-generating portions which must be empirically determined and subtracted. However, when each raw rate is properly converted, powerful conclusions can be reached, including what the rate of total cellular ATP production is, and how ATP production rate is divided between glycolysis and oxidative phosphorylation (28).

Existing measurements of ATP production demonstrate their value in understanding cancer cell energetics. Using the MCF7 epithelial, ER^+^ breast cancer cell line, Guppy et al. demonstrated that in contrast to prevailing models of high “aerobic glycolysis” by these cells, as commonly extrapolated from the Warburg effect and later applied to the “addiction” of cancer cells to glutamine and glucose, these cells were primarily oxidative and derived less than half of their ATP from either glucose or from glutamine under the conditions assayed (29).

Recent instrumentation advances have greatly simplified the measurements of ATP production-associated rates in a way that makes further elucidation of cancer cell bioenergetics, specifically ATP production and consumption, much more straightforward. The Agilent XF Analyzer, which measures simultaneous O_2_ and H^+^ fluxes, is one example. However, as mentioned above, these extracelular flux rates cannot be used to draw conclusions about total cellular ATP production without correction and conversion to ATP flux (J_ATP_).

We have developed a method for performing the correction and conversion needed to calculate rates of glycolytic ATP production (J_ATPglyc_) and oxidative ATP production (J_ATPglyc_) rate from all intracellular sources, allowing their summation to total cellular ATP production (J_ATPproduction_) as well as determination of the proportional contributions of each ATP-generating pathway as summarized in the Materials and Methods and in Mookerjee et al. (28). Here, we apply this method to re-examine the bioenergetic behavior of MCF7 cells in culture. We investigate their ability to use different common extracellular substrates to meet energy demand, and to determine how flexibly these cells can meet that demand specifically by shifting between glycolysis and oxidative phosphorylation. In addition to flexibility of ATP supply, we also demonstrate that MCF7 cells display flexibility in the substrate-driven and maximum rates of ATP production supported by different substrate conditions. Finally, we demonstrate that increased ATP supply through recruitment of substrate oxidation is the likely driver of bioenergetic variation in MCF7 cells.

## Materials and Methods

### Reagents

Chemicals (substrates, drugs, assay medium components) were from Sigma-Aldrich (St. Louis, MO) unless otherwise noted. Cell culture reagents were from Corning (Bedford, MA). Seahorse XF consumables (cartridges, cell culture plates) were from Agilent (Santa Clara, CA). The bicinchoninic (BCA) assay for protein, tetramethylrhodamine methyl ester (TMRM) and other fluorescence probes were from Life Technologies (Carlsbad, CA). The FLIPR Membrane Potential Assay Explorer Kit was from Molecular Devices (Sunnyvale, CA; #R8126 red version). Zosuquidar was from Cayman Chemical (Ann Arbor, MI).

### Cell Culture

MCF7 cells (ATCC) and mouse C2C12 myoblasts (ATCC) were cultured in Dulbecco's modified Eagle's medium (Corning #10-013) with added 10% (v/v) fetal bovine serum (FBS), 100 units/ml penicillin, and 100 μg/ml streptomycin. Cells were analyzed within 1 year of purchase from ATCC.

### Seahorse XFe96 Assay and Analysis

Twenty four to forty eight hours before the assay, adherent cells were plated in 100 μl culture medium at 10,000–12,000 cells/well in the inner 60 wells of a 96-well polystyrene Seahorse V3-PS Flux plate with no additional coating. Culture medium was added to the remaining outer wells. Twenty five minutes before the assay start, all wells were washed three times and then incubated in 180 μl of Krebs-Ringer phosphate HEPES (KRPH) medium [2 mM HEPES, 136 mM NaCl, 2 mM NaH_2_PO_4_, 3.7 mM KCl, 1 mM MgCl_2_, 1.5 mM CaCl_2_, 0.1% (w/v) fatty-acid-free bovine serum albumin, pH 7.4 at 37°C]. This starvation protocol is consistent with our prior work and similar to others (30) and within the measurement error, measured rates are consistent with the assumption of exclusive catabolism of added substrates (28). At the start of the assay, medium was replaced with 180 μl fresh KRPH at 37°C. All assays were carried out at 37°C at a starting pH of 7.4. Cell respiratory control (31) and glycolytic capacity (32) were assayed by measuring oxygen consumption and extracellular acidification rates in a Seahorse XFe96 extracellular flux analyzer as previously described (28). Cell respiratory control was assessed by the addition via ports A–D of either 10 mM glucose, 10 mM pyruvate, 2 mM glutamine, or a mix of all three in A, followed in all cases by 2 μg/ml oligomycin in B, 0.5–1 μM FCCP [carbonyl cyanide 4-(trifluoromethoxy)phenylhydrazone] in C, and 1 μM rotenone plus 1 μM myxothiazol in D. To minimize FCCP toxicity dampening maximum respiration rate, FCCP was carefully titrated in both cell lines prior to performing experiments. 0.5 μM was used for MCF7 cells while 1 μM was used for C2C12 cells. To minimize the potential for energetic collapse dampening maximum respiration rate after oligomycin addition, we conducted separate experiments where oligomycin and FCCP were added simultaneously. The highest rate of maximum respiration achieved in the presence of oligomycin and FCCP, whether sequential or simultaneous, was used to calculate maximum J_ATPox_. Substrate concentrations were close to common concentrations in cell culture and consistent with our previous analyses (32, 33).

Glycolytic capacity was assessed with the same substrate additions as for cell respiratory control in port A, 1 μM rotenone plus 1 μM myxothiazol in B, and 100 μM monensin in C. All measurement cycles consisted of a 1 min mix, 1 min wait, and 3 min rate measurement. Three measurement cycles were performed prior to any addition, 6 cycles after the port A substrate addition, and 3 cycles after all subsequent additions. Assay duration was ~75 min. Following the assay, the cell-containing plate wells were washed three times with 100 μl PBS (137 mM NaCl, 2.7 mM KCl, 10 mM Na_2_HPO_4_, 1.8 mM KH_2_PO_4_, pH 7.4 @ 22°C). 10 μl 5.0 % (w/v) sodium deoxycholate was added to each well and plates were agitated on a plate shaker at 800 rpm for 5 min to lyse cells. Thirty μL water was then added, and 10 μL of the resulting sample from each well was analyzed by BCA assay for total protein content calibrated to a BSA standard. Protein values (μg/well) were used to normalize the rates of oxygen consumption and extracellular acidification in each well.

The Wave software native to the XF Analyzer was used to extract rates of oxygen consumption and extracellular acidification.

### Calculation of ATP Production Rate (J_ATPproduction_)

We have previously described a method for calculation of total cellular ATP production rate from extracellular acidification and oxygen consumption rates (28). This calculation fully accounts for net ATP production in the cell, including net ATP production in glycolysis (at phosphoglycerate kinase and pyruvate kinase, minus consumption at hexokinase and phosphofructokinase), the TCA cycle (at succinyl CoA synthetase), and by the mitochondrial ATP synthase. A brief description of the calculation and its underlying assumptions is presented here.

To calculate J_ATPglyc_, the extracellular acidification rate was first converted to total proton production rate. The contribution of respiratory CO_2_ to total proton production rate was subtracted to yield glycolytic rate of glucose catabolism terminating in lactate. This rate was multiplied by the ratio of ATP produced in glycolysis terminating in lactate per extracellular H^+^ (the P/H^+^ ratio). Additional glycolytic flux generating the pyruvate that is later fully oxidized in the mitochondria generates additional ATP, and is represented in the mitochondrial respiration rate (see below). Therefore, mitochondrial respiration rate was multiplied by the ratio of ATP produced in glycolysis terminating in pyruvate per O_2_ consumed for each substrate (P/O_glycolysis_). Glycolytic ATP production (J_ATPglyc_) was calculated as the sum of these two rates.

To calculate J_ATPox_, mitochondrial respiration rate was isolated by subtracting from the total oxygen consumption rate any additional oxygen consumption in the presence of mitochondrial poisons rotenone and myxothiazol. Mitochondrial respiration rate was further divided into ATP-coupled and uncoupled respiration rates using the mitochondrial ATP synthase inhibitor oligomycin. The ATP-coupled respiration rate was multiplied by the portion of the P/O ratio attributable to the mitochondrial ATP synthase (P/O_oxphos_). To account for oxidative substrate-level phosphorylation in the TCA cycle, the mitochondrial respiration rate was multiplied by the P/O ratio attributable to succinyl CoA synthetase (P/O_TCA_). Oxidative ATP production (J_ATPox_) was calculated as the sum of these two rates.

Finally, J_ATPglyc_ and J_ATPox_ were summed to yield the total cellular ATP production rate, J_ATPproduction_. Note that when FCCP (or another mitochondrial uncoupler) is present and respiration is fully uncoupled, J_ATPox_ is theoretical, as actual oxidative phosphorylation does not occur under these conditions. Though the biological meaning of the maximum respiration rate achievable in a cell with the addition of an uncoupler is not clear, it is generally interpreted as the theoretical capacity of oxidative phosphorylation (31).

The biological assumptions of the ATP calculation model include exclusive use of exogenous substrates, proportional use of single substrates when substrate combinations are given, complete oxidation of these substrates (for glutamine, partial oxidation to lactate), transport of reducing equivalents into the mitochondrial matrix primarily by the malate-aspartate shuttle, and negligible cell growth under the conditions and timeframe assayed. Technical assumptions and calibration of the XF Analyzer measurements have been described previously (34, 35).

### Mitochondrial Membrane Potential Measurement and Analysis

Absolute mitochondrial membrane potential (ΔψM) was measured by fluorescence microscopy as previously described (36, 37). Briefly, MCF7 cells were plated in 100 μl culture medium in Matrigel-coated glass-bottom 96-well culture plates at 10,000 cells/well 48 h prior to the experiment. Three hours before recording, cells were washed twice with a modified culture medium containing TMRM (non-fluorescent DMEM with 10% FBS, 4.5 mg/L glucose, 1 mM NaHCO_3_, 20 mM TES, 1 mM pyruvate, 4 mM glutamine, 10 nM TMRM) in an air incubator at 37°C in order to equilibrate the probe. Next, the recording was set up (2 positions/well and 18 wells per plate), and then cultures were washed 3× with a Potentiometric Medium (PM) closely resembling the assay medium used for J_ATPglyc_ and J_ATPox_ determinations (KRPH; 10 nM TMRM, 1:100 FLIPR, 1 μM tetraphenylborate, 1 μM zosuquidar) with no substrates. In order to keep constant probe concentration during the assay, all media and substrates added to the cultures were prepared by 1:1 mixing of a common 2×PM (with 32 mM NaCl) with 240 mM NaCl (resulting PM), or aqueous stocks of substrates. Subsequently, after 10 min wait, a baseline recording of 30 min was started, followed by 30-min segments of substrate additions. Imaging was performed on a Nikon Eclipse Ti Perfect Focus System fully motorized wide-field fluorescence microscope and equipped with a custom Lambda 821 LED light source (Sutter Instruments, Novato, CA) with Lambda 10-3 emission filter wheel an Andor iXon Life 888 EMCCD camera (Oxford Instruments, UK) and a Nikon motorized stage with Elements 5.20 (Nikon, Melville, NY) using an S-Fluor 20× air lens. TMRM and FLIPR signals were collected at 100 s intervals, using the following filter sets, given as LED nm, excitation—emission in nm/bandwidth, for TMRM: 561, 586/20–641/75 (30 ms exposure time, 14% power) and for FLIPR: 506, 509/22–542/27, using a 459/526/596 beamsplitter (30 ms, 7%; all from Semrock, Rochester, NY). Recordings were analyzed in Image Analyst MKII (Image Analyst Software, Novato, CA) using the “Mitochondrial membrane potential assay (TMRM/PMPI) with masking dead cells” standard pipeline. ΔψP and ΔψM were calibrated using the “Complete Iterative” and “Complete (known k)” paradigms, respectively, with k_T_ = 0.01 s^−1^. To this end, two calibrants were used; first ΔψM was completely depolarized using 1 μM oligomycin, 1 μM FCCP, 4 μM antimycin A, 4 μM myxothiazol and 1 μM valinomycin (a K^+^-ionophore) in PM and the decay in TMRM fluorescence was recorded immediately for 30 min. This was followed by complete depolarization of the ΔψP by adding PM containing 2% paraformaldehyde, 120 mM KCl, and 5 μg/mL gramicidin (a Na^+^ ionophore). The upstroke of the FLIPR fluorescence intensity upon this calibration step was used as “Part complete ΔψP depolarization,” and each cell in each viewfield was independently calibrated. Changes in ΔψM after exogenous substrate addition were calculated and analyzed using GraphPad Prism and Microsoft Excel.

### Statistical Analysis

Statistical analysis was performed in GraphPad Prism. Welch's *t*-test (no assumption of equal standard deviation) was performed for all pairwise comparisons as described in the figure captions. For multiple comparisons, ordinary one-way ANOVA followed by Tukey's *post-hoc* multiple comparisons test was performed as described in figure captions. Technical replicate error (of 3–6 wells/plate) was discarded. Error bars for all values represent the standard error of the mean of 4–6 independent experiments except where noted.

## Results

### Mixed Substrates Drive Higher Rates of Total Cellular ATP Production (J_ATPproduction_) in MCF7 Cells

**Figure 1:**
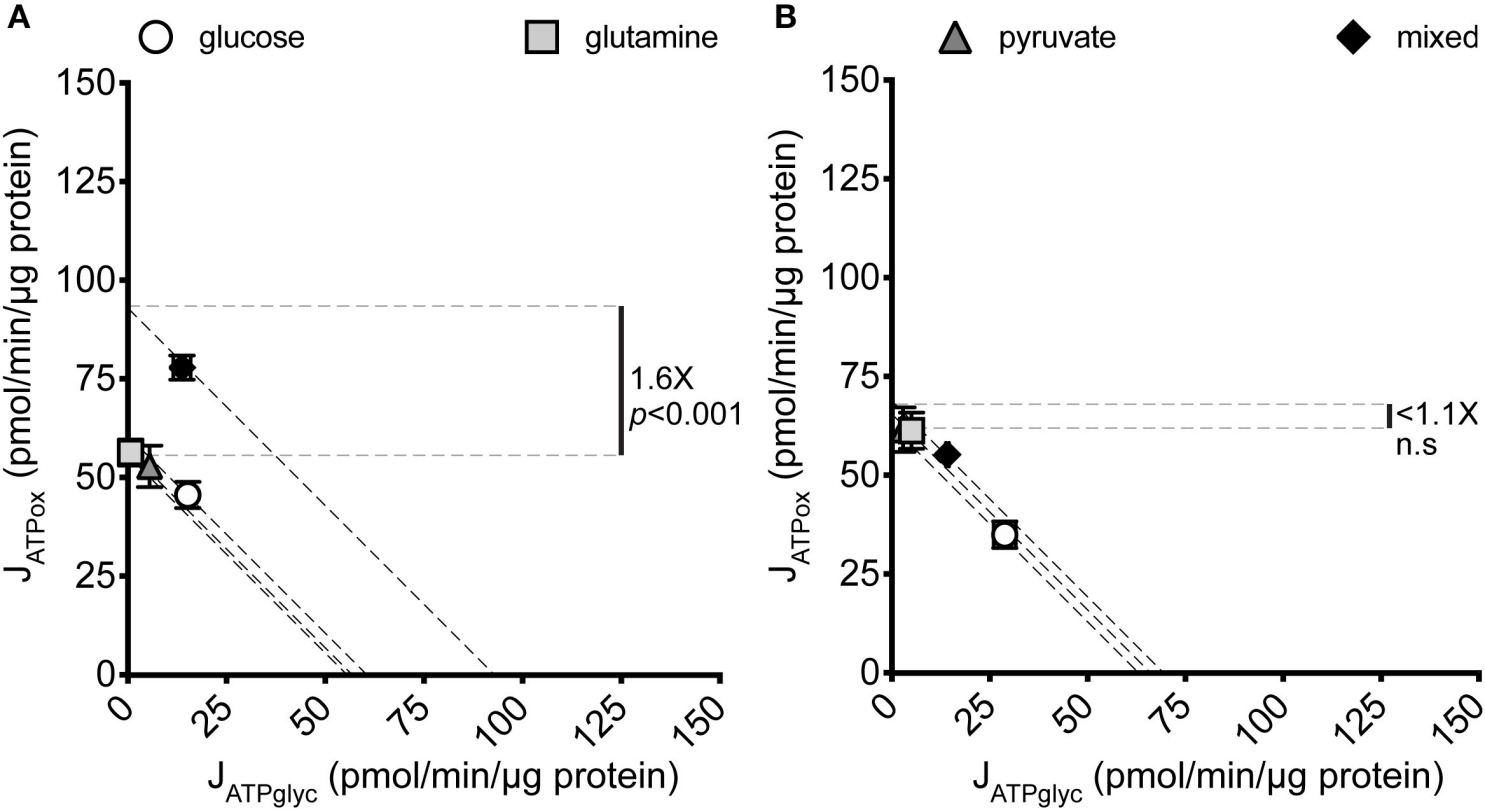
J_ATPproduction_ of MCF7 and C2C12 cells under different substrate conditions. Glycolytic ATP production rate (J_ATPglyc_) and oxidative ATP production rate (J_ATPox_) are plotted on the *x* and *y* axes, respectively. Each data point represents the mean of 4–6 independent experimental replicates ± SEM. Independent replicates are the average of 3–6 technical replicates per plate. Statistical comparisons were made using ordinary one-way ANOVA with Tukey's multiple comparisons test. Dashed diagonal lines represent all possible distributions of J_ATPglyc_ and J_ATPox_ for the substrate-driven J_ATPproduction_ shown. **(A)** MCF7 cells. **(B)** C2C12 mouse myoblast cells.

To determine how J_ATPproduction_ in MCF7 cells changes with substrate provided, MCF7 cells cultured under standard conditions were briefly starved (25 min) to deplete endogenous substrates (30) and then assayed for extracellular flux of H^+^ and O_2_ in the minimal salts buffer KRPH. The extracellular substrates glucose, glutamine, and pyruvate, either singly or as a combination of all three, were added during the experiment. Figure 1A shows the significant variation in J_ATPproduction_ that results under the conditions assayed.

Glucose, glutamine, and pyruvate yielded similar J_ATPproduction_, of 63.4 ± 2.2, 56.7 ± 3.0, and 57.6 ± 1.8 pmol ATP/min/μg protein, respectively, with no statistically significant differences. When all three substrates were combined, J_ATPproduction_ increased significantly (*p* ≤ 0.001) to 91.6 ± 3.7 pmol ATP/min/μg protein. The highest substrate-driven rate achieved under the conditions tested (91.6 pmol ATP/min/μg protein, mixed substrates) was 1.6-fold higher than the lowest recorded substrate-driven J_ATPproduction_ of 56.7 pmol ATP/min/μg protein for pyruvate. The single substrates were likely being used by cells, as they supported respiration after the addition of FCCP in the cell respiratory control assays, while rates in cells receiving no exogenous substrate fell to zero, presumably through energetic collapse (not shown). The glycolytic index (GI) values for these substrate conditions demonstrate that substantial rates of J_ATPglyc_ only occurred when glucose was present (Table 1).

**Table 1:** Quantification of MCF7 bioenergetic phenotypes under different substrate conditions.

**MCF7 Bioenergetic Indices by Substrate in KRPH Assay Medium**				
	**Glucose**	**Glutamine**	**Pyruvate**	**Mixed**
GI (%)	23.6 ± 1.0	−0.1 ± 0.5	9.6 ± 0.3	15.0 ± 0.7
CI (%)	20.2 ± 0.9	–	–	12.2 ± 0.6
SFI (%)	100	30	33	100

The Glycolytic Index (GI) describes the percent of total cellular energy production that is produced by glycolysis; GI = 100^*^J_ATPglyc_/J_ATPproduction_. The Crabtree Index (CI) describes the degree to which cells will shift from oxidative to glycolytic ATP production when a glycolyzable substrate is introduced (or increased); CI = GI_condition2_ – GI_condition1_, where condition 1 is prior to substrate addition (here in the absence of exogenous substrates) and condition 2 is after this addition. Dashes denote conditions not applicable to CI measurement. The Substrate Flexibility Index (SFI) describes the range over which a cell can shift between J_ATPglyc_ and J_ATPox_ to maintain a particular J_ATPproduction_, limited by the maximum rates of glycolysis and respiration achievable under the conditions given. Each data point represents the mean of 4–6 independent experimental replicates ± SEM. Independent replicates are the average of 3–6 technical replicates per plate.

### Access to Mixed Substrates Does Not Consistently Increase J_ATPproduction_ in Cultured Cells

To ask whether the increased J_ATPproduction_ in MCF7 cells given multiple substrates occurs as a function of cell culturing, and is not intrinsic to these cells, we analyzed another cultured cell line under the same substrate conditions as the MCF7 cells. Figure 1B shows that the non-transformed C2C12 mouse myoblast line displayed no significant variation across the same single- and mixed-substrate conditions; for glucose, 60.1 ± 9.8; glutamine, 64.1 ± 6.5; pyruvate, 64.52 ± 6.4; mixed substrates, 68.9 ± 9.3. There were additionally no consistent trends in the C2C12 rates between individual experiments, suggesting no potential for small but meaningful differences between the substrate conditions as measured. The C2C12 measurements under glucose-only conditions were consistent with our previously published values for this cell line under the same conditions (28).

### Mixed Substrates Increase J_ATPproduction_ by Increasing J_ATPox_ in MCF7 Cells

**Figure 2:**
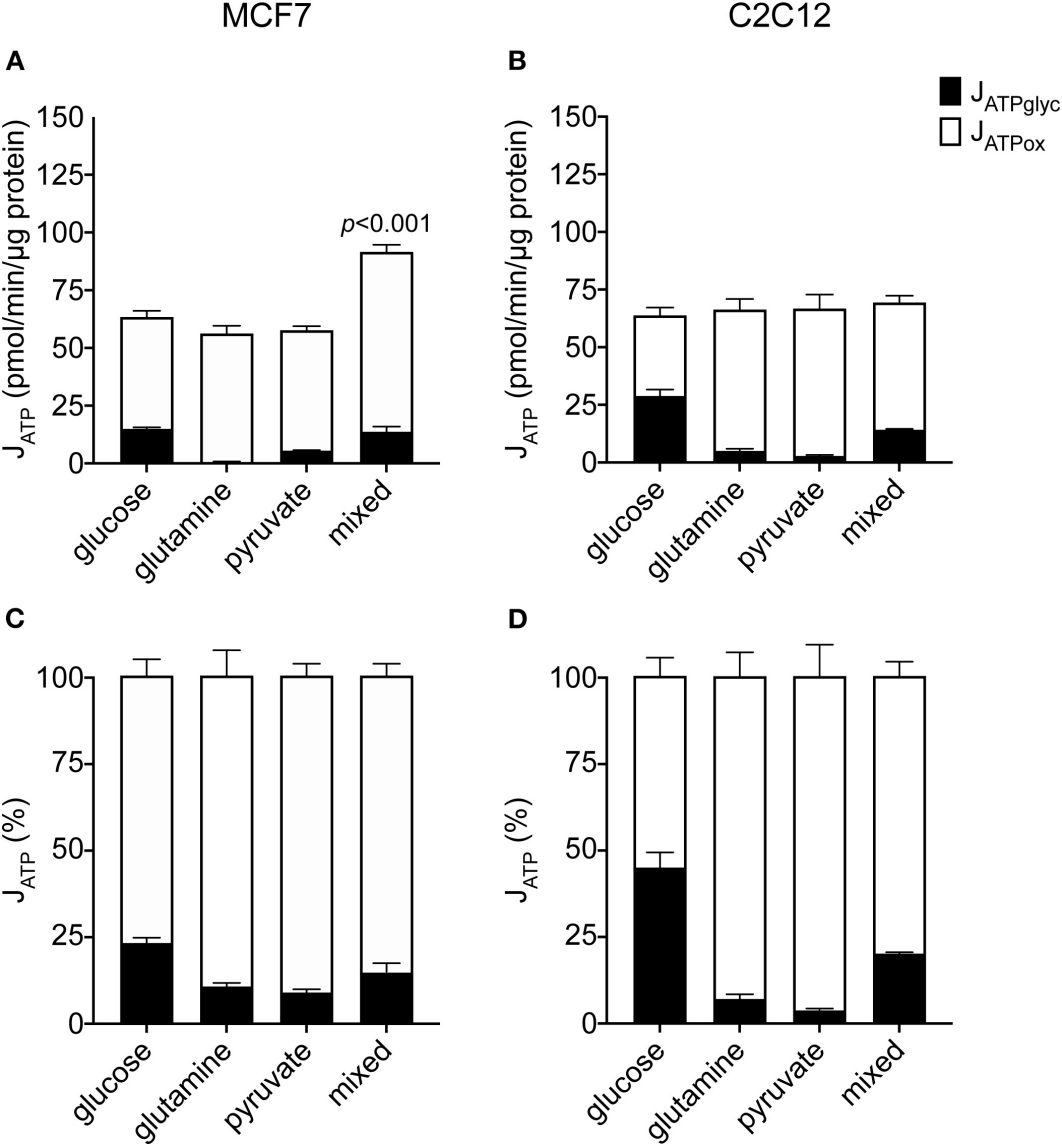
Absolute and proportional J_ATPglyc_ and J_ATPox_ in MCF7 and C2C12 cells. Absolute rates (pmol ATP/min/μg protein) from Figure 1 are displayed as stacked columns **(A,C)** and as percent of total J_ATPproduction_ **(B,D)**. Each column represents the mean of 4–6 independent experimental replicates ± SEM. Independent replicates are the average of 3–6 technical replicates per plate. Statistical comparisons of total J_ATPproduction_ were made using ordinary one-way ANOVA with Tukey's multiple comparisons test.

To understand how changes in J_ATPproduction_ occur in the MCF7 cells under different substrate conditions, we examined the constituent rates of J_ATPglyc_ and J_ATPox_ in the same dataset shown in Figure 1A. Figures 2A,B show the composition of J_ATPproduction_ in absolute units, while Figures 2C,D show the proportional contributions of each pathway in the MCF7 and C2C12 lines, respectively. Glycolytic ATP production in the absence of exogenous substrates was 1.6 ± 0.3 pmol ATP/min/μg protein (not shown), and changes negligibly with the addition of glutamine or pyruvate, as expected for these non-glycolyzable substrates. While pyruvate could be reduced to lactate, the charge of each molecule is the same and therefore no net acidification would occur. No significant changes occur in J_ATPox_ after the addition of glutamine or pyruvate individually. J_ATPproduction_ and proportional distribution between J_ATPglyc_ and J_ATPox_ were comparable between these cell lines.

In contrast to the single substrates glutamine and pyruvate, glucose, either alone or combined with glutamine and pyruvate, recruits glycolysis and drives J_ATPglyc_ (Figures 2A,B). The percentage J_ATPglyc_ is the cell's glycolytic index (GI) value (Figures 2C,D, Table 1) under the stated conditions. Since glycolysis requires a glycolysis substrate, J_ATPglyc_ would be expected only from a sugar, e.g., glucose, and the GI index value should be zero. For glutamine, this is true within the error of the calculation. For pyruvate, there was a small non-zero rate, which may represent a source of acidification not accounted for in the calculation model or an overestimate of the total proton production rate.

Glucose addition and subsequent J_ATPglyc_ was accompanied by a roughly equal decrease in J_ATPox_, resulting in no net change to J_ATPproduction_. The illustrates the Crabtree effect (17), and is quantified in Table 1 as the Crabtree index. When glucose was the sole substrate, the decrease in J_ATPox_ was balanced by a roughly equal increase in J_ATPglyc_, so that J_ATPproduction_ did not change, and was not significantly different from the rates conferred by the other single substrates. However, combination of all three substrates allowed recruitment of J_ATPglyc_ similar to glucose alone, as well as a substantial increase in J_ATPox_, to yield an overall increase in J_ATPproduction_ in the MCF7 cells.

### Glutamine, but Not Pyruvate, Confers Additional Oxidative Capacity in MCF7 Cells

**Figure 3:**
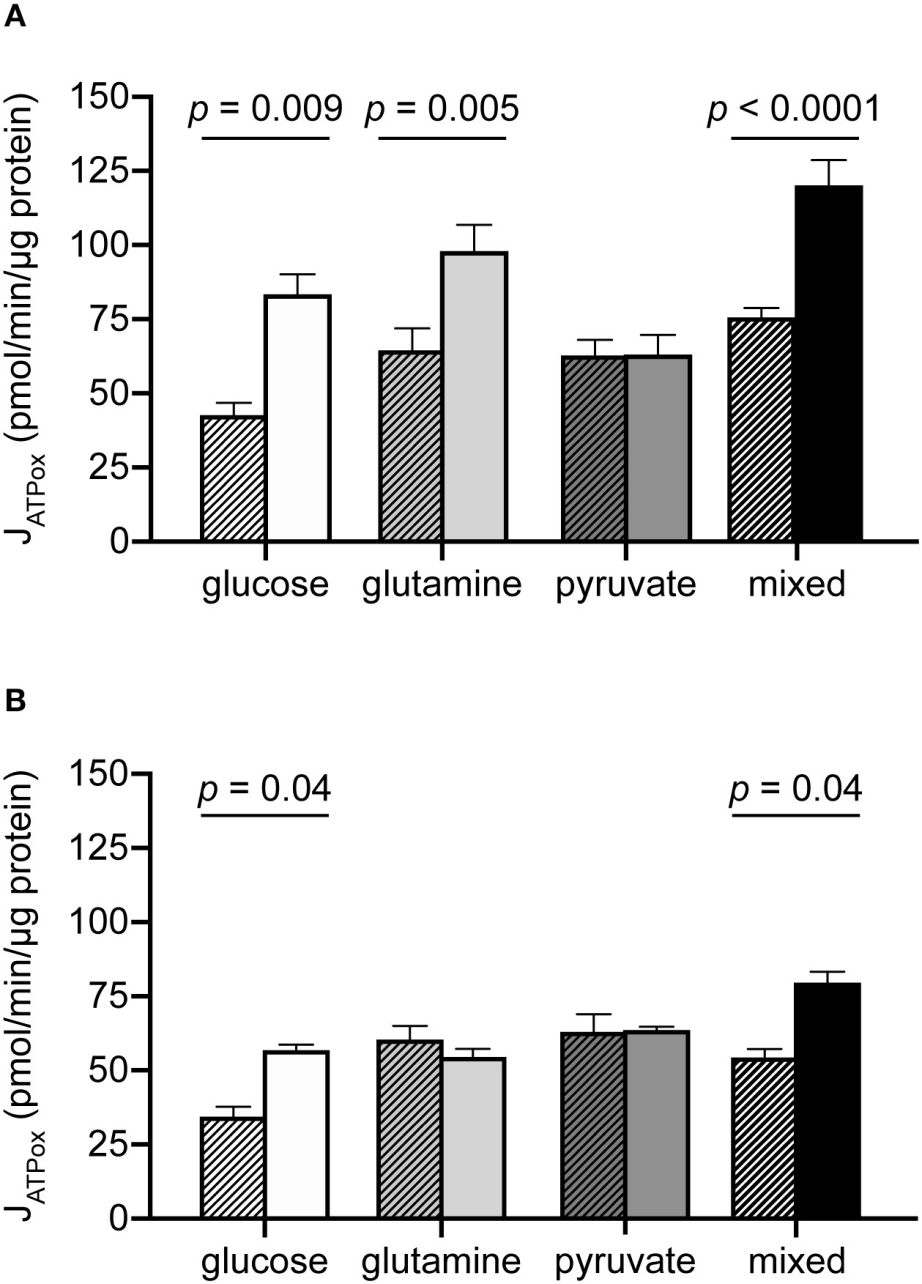
Additional oxidative capacity conferred by glutamine but not pyruvate in MCF7 cells. J_ATPox_ for MCF7 **(A)** and C2C12 **(B)** under substrate-driven (hatched bars) and maximum respiration-eliciting conditions for all substrate conditions assayed. Each column represents the mean of 4–6 independent experimental replicates ± SEM. Independent replicates are the average of 3–6 technical replicates per plate. Statistical comparisons were made using ordinary one-way ANOVA with Tukey's multiple comparisons test.

The results above are consistent with an expectation that glutamine and pyruvate will support similar bioenergetic rates, as both are respiratory substrates. However, while the J_ATPproduction_ resulting from their individual use was similar, a few notable differences emerged when cells were driven to their oxidative capacities under each condition (Table 2, Figure 3). The addition of the mitochondrial uncoupler FCCP to provoke maximum respiration did not increase pyruvate-driven respiration past its substrate-driven rate, suggesting that pyruvate was being used at its capacity under the conditions measured. In the presence of glutamine, however, MCF7 cells were able to increase theoretical J_ATPox_ (as a function of respiration) 1.75-fold above its initial rate (Figure 3A).

**Table 2:** Maximum rates and bioenergetic capacities in MCF7 under different substrate conditions.

**Maximum rates of J_ATPproduction_ in MCF7 cells in KRPH assay medium (pmol ATP/min/μg protein)**				
	**Glucose**	**Glutamine**	**Pyruvate**	**Mixed**
J_ATPglycmax_	149.8 ± 13.8	23.0 ± 4.3	27.0 ± 3.8	154.5 ± 7.8
J_ATPoxmax_	83.4 ± 6.8	98.5 ± 5.7	52.1 ± 1.8	120.2 ± 8.6
Bioenergetic capacity	233.2 ± 15.4	121.5 ± 7.1	79.1 ± 4.1	274.7 ± 11.5

*Maximum rates of J_ATPglyc_ and J_ATPox_ were determined as described in Methods and shown in Figure 5. The theoretical bioenergetic capacity is the sum of these values. Each data point represents the mean of 4–6 independent experimental replicates ± SEM. Independent replicates represent the average of 3–6 technical replicates per plate*.

C2C12 cells did not appear to utilize glutamine to access higher rates of J_ATPox_ (Figure 3B). Glutamine-driven J_ATPox_ in C2C12 cells did not significantly change with FCCP addition to drive maximum respiration under the experimental conditions tested. Pyruvate-driven J_ATPox_ was also unable to increase when driven to its theoretical capacity by FCCP. For glucose and mixed substrates, oxidative capacity was significantly higher than substrate-driven rates.

### Pyruvate, but Not Glutamine, Stimulates Increased Substrate-Driven J_ATPproduction_ by Increasing J_ATPox_

**Figure 4:**
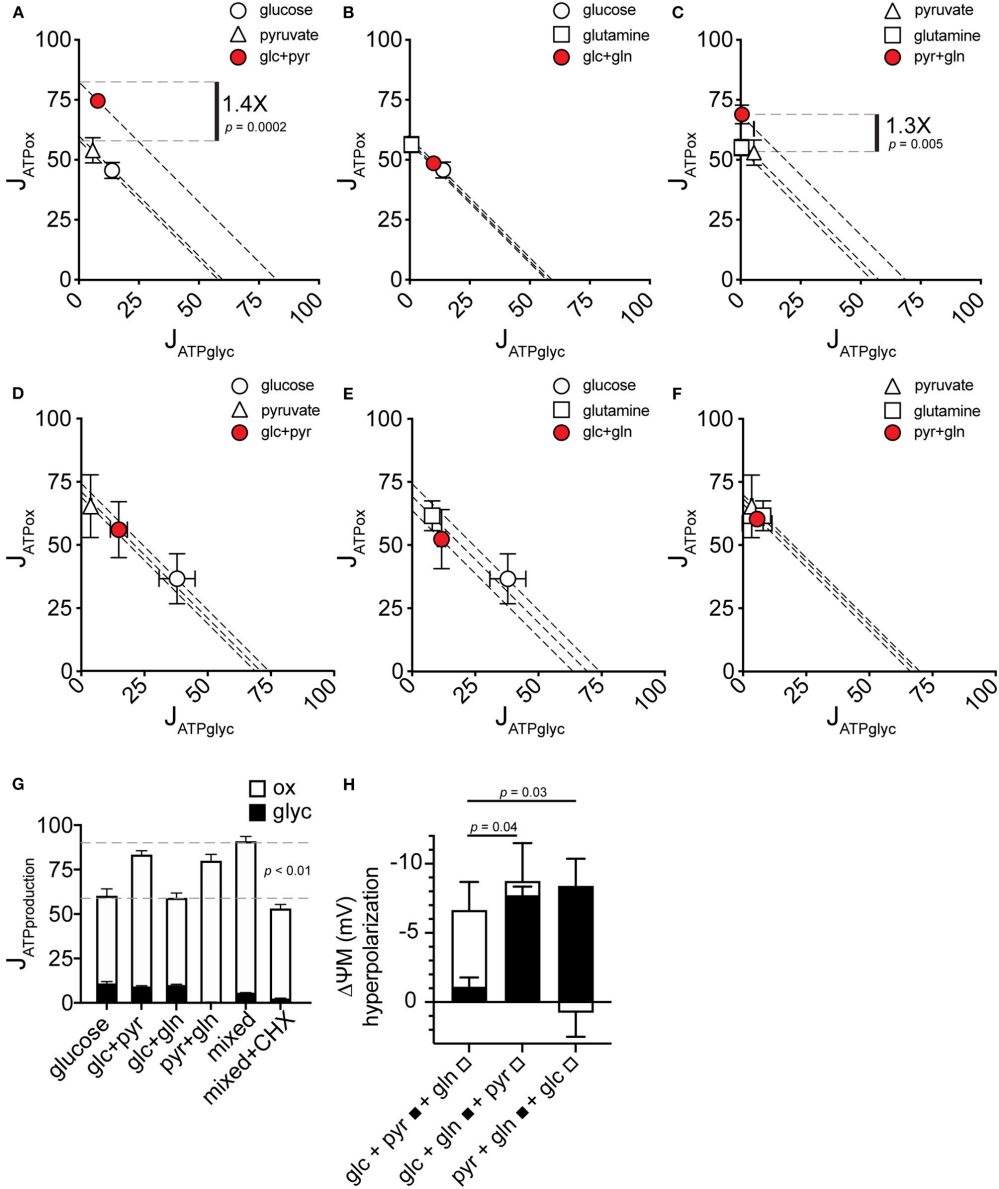
J_ATPproduction_ in pairwise substrate combinations. **(A–F)** Glycolytic ATP production rate (J_ATPglyc_) and oxidative ATP production rate (J_ATPox_) are plotted on the *x* and *y* axes, respectively, for MCF7 **(A–C)** and C2C12 **(D–F)** cells catabolizing the substrates indicated. Open symbols indicate single substrates; filled symbols indicate substrate pairs. Each data point represents the mean of 4–6 independent experimental replicates ± SEM. Independent replicates are the average of 3–6 technical replicates per plate. Statistical comparisons were made using ordinary one-way ANOVA with Tukey's multiple comparisons test. Dashed diagonal lines represent all possible distributions of J_ATPglyc_ and J_ATPox_ for the substrate-driven J_ATPproduction_ shown. **(G)** MCF7 J_ATPproduction_ from substrate pairs with comparison to mixed substrates and to mixed substrates plus 10 μM cycloheximide (CHX). glc, glucose; pyr, pyruvate; gln, glutamine. **(H)** Change in ΔψM after addition of the indicated substrates. A pair of substrates (black bars) was followed by the addition of the third substrate (white bars). Data are mean ± SEM of *n* = 3 independent experimental replicates.

To resolve how the mixed-substrate condition confers higher J_ATPproduction_ in MCF7, we carried out a pairwise analysis of all added substrates. We found that pyruvate, in any combination, was associated with increased J_ATPproduction_ similar to the mixed-substrate condition (Figures 4A,C). Curiously, pyruvate alone did not confer this effect, supporting the same J_ATPproduction_ as each substrate alone (Figure 1A). Cells using glucose plus glutamine displayed substrate-driven J_ATPproduction_ no different from either substrate alone (Figure 4B). Similar to Figure 1, the same analysis of paired substrates in C2C12 cells revealed no significant differences in J_ATPproduction_ between any substrate pair and each single substrate (Figures 4D–F). Notably, while glucose alone induced the highest J_ATPglyc_ (almost 50% of J_ATPproduction_), substrate pairs containing glucose shifted to intermediate positions along the “iso-J_ATP_” line denoting the same J_ATPproduction_ along different proportional contributions of J_ATPglyc_ and J_ATPox_, further supporting the assumption of exogenous substrate catabolism by the cells. J_ATPproduction_ for each substrate pair is plotted in Figure 4G, together with the mixed-substrate J_ATPproduction_. The last column shows that J_ATPproduction_ was sensitive to decreased ATP demand caused by inhibiting protein synthesis using cycloheximide.

### Glutamine Allows Access to Additional Oxidative Capacity in MCF7 Cells by Increasing ATP Supply Through Substrate Oxidation

To test possible mechanisms of the increase in J_ATPox_ conferred by substrate combinations containing pyruvate, we measured mitochondrial membrane potential in MCF7 cells under the conditions used for extracellular flux analysis. An increase in cellular J_ATPox_ requires an increase in total activities of substrate oxidation, e.g., by recruitment of additional substrates to feed ATP supply, or an increase in ATP consumption by additional ATP demand. These supply and demand reactions are linked by the ΔψM as a common intermediate (31), which will increase (hyperpolarize) if increased J_ATPox_ results from faster substrate oxidation. Conversely, ΔψM will decrease (depolarize) if increased J_ATPox_ results from increased ATP demand. Glutamine hyperpolarized ΔψM when it was added to MCF7 cells in the presence of the other two substrates (Figure 4H). This indicates an increased activity of substrate oxidation pathways when glutamine oxidation is recruited, and this may explain the observed increase in oxidative capacity in the presence of glutamine (Figure 3A).

### Pyruvate Increases J_ATPox_ by Increasing Both Supply and Cellular ATP Demand

In contrast to glutamine, pyruvate added to the other two substrates did not change ΔψM (Figure 4H). In light of the increased J_ATPproduction_ with pyruvate (Figures 4A–C), pyruvate likely independently increased the activities of both supply and demand. Direct stimulation of ATP demand by pyruvate is further supported by the finding that the ΔψM hyperpolarization with glutamine did not increase ATP turnover, suggesting that a change in supply activities is not sufficient to alter ATP demand, and further that control of overall energy metabolism was largely by ATP demand.

### MCF7 Supply Flexibility and Bioenergetic Capacity Changes With Substrate Availability

**Figure 5:**
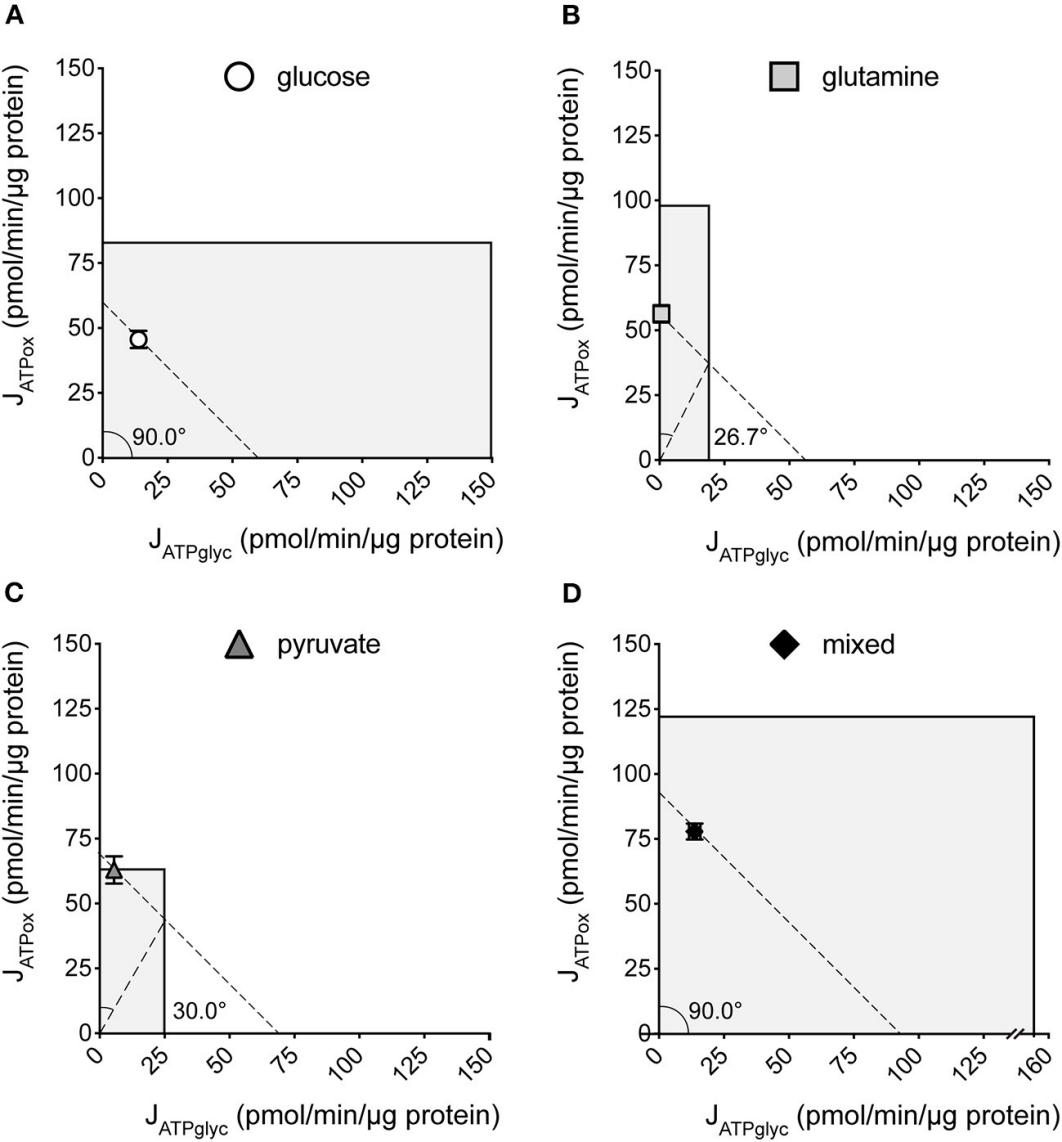
Bioenergetic capacities and substrate flexibilities in MCF7 cells. Maximum J_ATPox_ and J_ATPglyc_ determined as described (Methods) for cells catabolizing exogenous glucose **(A)**, glutamine **(B)**, pyruvate **(C)**, or a mix of all three **(D)**. Boundaries of the shaded boxes represent theoretical (J_ATPox_) or actual (J_ATPglyc_) capacities, with their x,y intersection the theoretical “bioenergetic capacity” afforded under each tested condition. Shaded area within each box is the theoretical “bioenergetic space,” or all possible values of J_ATPglyc_ and J_ATPox_ that the cell could achieve under experimental conditions. Angular notation illustrates supply flexibility for the substrate-driven J_ATPproduction_ shown. Dashed diagonal lines represent all possible distributions of J_ATPglyc_ and J_ATPox_ for the substrate-driven J_ATPproduction_ shown, within the bioenergetic capacities represented by the *x*- and *y*- boundaries of the shaded box. Each point and capacity represents the mean of 4–6 independent experimental replicates ± SEM. Independent replicates are the average of 3–6 technical replicates per plate.

We previously described how substrate-driven J_ATPproduction_ could be analyzed within the context of cellular bioenergetic capacity [Figure 5, (28)] to assess the theoretical flexibility of cellular J_ATPproduction_ to increase, decrease, and shift between J_ATPglyc_ and J_ATPox_. We apply that analysis to the MCF7 cells examined here. Figures 5A–D show the substrate-driven J_ATPproduction_ for a different substrate condition; the single substrates glucose (A), glutamine (B), and pyruvate (C), and the mixed-substrate condition containing all three (D). Each panel shows a shaded area representing the theoretical bioenergetic activity in the cells under each condition. The theoretical maximum J_ATPox_, as calculated from the respiratory rate after mitochondrial uncoupling with FCCP in the presence of oligomycin, forms the horizontal boundary intersecting the *y*-axis. Maximum J_ATPglyc_, determined by adding the Na^+^-ionophore monensin in the presence of rotenone and myxothiazol (but not oligomycin, which will inhibit ATP hydrolysis by the mitochondrial ATP synthase), forms the vertical boundary intersecting the *x*-axis. Using this convention, we assessed the ATP supply flexibility of the cell, that is, how flexibly the cell can meet a given ATP demand by drawing on J_ATPglyc_ and J_ATPox_ before it reaches the capacity of either production pathway.

ATP supply flexibility was quantified using the Supply Flexibility Index [SFI (28), Table 1]. As shown in Figure 5 and Table 1, MCF7 cells catabolizing glucose displayed a J_ATPproduction_ of 63.4 ± 2.2 pmol/min/μg protein, with 23.6% of the total J_ATPproduction_ derived from glycolysis. J_ATPglyc_ and J_ATPox_ were both below their individual maxima (149.8 ± 13.8 and 83.4 ± 6.8, respectively), and the J_ATPproduction_ itself was about half of the theoretical bioenergetic capacity (sum of the two maxima) of 233.2 ± 15.4 pmol ATP/min/μg protein. To maintain substrate-driven J_ATPproduction_, the cell therefore had access to a wide range of its apparent bioenergetic capacity. ATP supply flexibility was quantified from the angle formed by lines extending from the origin to the intersection of the iso-J_ATP_ line with the ATP production capacity under the specified conditions. In cells catabolizing glucose (Figure 5A), the iso-J_ATP_ line extends to each axis without intersecting either capacity, so that the angle measured at the axis is 90°. The substrate flexibility of the MCF7 cells under these conditions was therefore 100^*^90/90 = 100%. In other words, MCF7 cells using glucose had access to 100% of the possible distribution between J_ATPglyc_ and J_ATPox_ to meet ATP demand. In contrast, glutamine should confer no glycolytic capacity and therefore an SFI of zero. The small calculated J_ATPglyc_ that does appear might represent extracellular acidification in the presence of monensin that is not accounted for in the calculation model. The calculated SFI of 100^*^26.7/90 = 30% is therefore likely an overestimate of ATP supply flexibility under these conditions. Similarly, the calculated SFI of 100^*^30/90° = 33% for cells oxidizing pyruvate is also likely to be an overestimate of ATP supply flexibility. Finally, under the mixed substrate condition, because it contains glucose, maintenance of substrate-driven J_ATPproduction_ was possible with any combination of J_ATPglyc_ and J_ATPox_, yielding an SFI of 100%.

## Discussion

We report here that MCF7 cells display considerable bioenergetic flexibility, demonstrated by their ability to support the same substrate-driven J_ATPproduction_ with different individual substrates (Figure 1), each with a different catabolic entry point, and in their ability to support substrate-driven J_ATPproduction_ with any proportional distribution of J_ATPglyc_ and J_ATPox_ (Figure 5). We find that this flexibility was not unique to malignant cells, as non-malignant C2C12 myoblasts, which behave similarly with respect to J_ATPproduction_, displayed similar flexibilities. However, MCF7 used its given substrates differently than C2C12 cells, particularly at high J_ATPproduction_. The higher J_ATPproduction_ associated with mixed substrates (Figure 1) appeared to be driven by pyruvate under substrate-driven conditions (Figure 4), and by glutamine when maximum respiration was elicited (Figure 5).

Interestingly, the comparison between these two cell lines shows that when provided a glycolysis substrate, MCF7 cells derived only a small proportion of its total cellular ATP from glycolysis (about 20%) even when glucose was the sole exogenous substrate, while the non-malignant C2C12 line was more glycolytically active both as a proportion of the total rate (about 50%) and in absolute glycolytic rate, despite similar J_ATPproduction_ to MCF7. The proportional assignment of J_ATPglyc_ and J_ATPox_ in MCF7 exactly matched prior determinations of these ratios made by measuring partial pressures of O_2_ and CO_2_ in closed glass culture chambers (29). This recapitulation supports the validity of applying our ATP calculation model to MCF7 cells (28).

These data also demonstrate the error of assuming that glycolysis produces all or most ATP in cancer cells, a common extrapolation of the Warburg effect. The Warburg effect is a useful observation as applied to e.g., tumor detection *in vivo* using positron tomography, but cannot be similarly applied to characterizing cancer cells *in vivo* or in cell culture, or to understanding their bioenergetic behavior.

The 1.6-fold, or 60% increase between the highest and lowest J_ATPproduction_ by MCF7 represents a substantial range of substrate-driven flux. The main driver of this increase appeared to be pyruvate oxidation, in combination with either glucose or glutamine but not alone. Pyruvate-driven increases in J_ATPox_ appeared to be due to roughly equal increases in ATP supply and ATP demand, as ΔψM hyperpolarization was relatively small (~1 mV, Figure 4H). In contrast, the presence of glutamine in any combination triggered a much more substantial hyperpolarization (~7 mV) indicating faster ATP supply. Glutamine-driven ΔψM hyperpolarization did not consistently correspond to faster J_ATPox_ (Figure 4B). Because glutamine did not increase J_ATPproduction_ (Figure 4B), despite the ΔψM hyperpolarization, it is possible that ΔψM has a weak control over ATP demand, i.e., higher ATP/ADP in the presence of more negative ΔψM does not drive demand reactions faster, so J_ATPox_ does not show a net increase.

Additionally, while the substrate-driven rates in both cell types supported by glutamine alone or by pyruvate alone were all similar, their oxidative capacities diverged considerably, with glutamine conferring substantially higher rates than pyruvate. One possible explanation for pyruvate's lack of ability to support additional capacity across both cell lines is artifactual; FCCP-mediated ΔψM collapse to drive maximum respiration rate could selectively slow H^+^/pyruvate symport into the mitochondrial matrix. If so, pyruvate-mediated J_ATPox_ might be higher with intact membrane potential but not detectable under our experimental conditions.

For glutamine, the different responses of C2C12 and MCF7 could represent differences in the architecture of glutamine oxidation between the two cell types. This observation may explain at least in part why a cancer cell might appear to be “glutamine-addicted,” if glutamine withdrawal deprives cells of access to a portion of its capacity that cannot be accessed by pyruvate or other substrates. Prior work on the iBMK cell line supports a substantial contribution of glutamine oxidation to ATP production (38), though this may vary by cell type (29). Further, as uncoupled respiration is controlled primarily by substrate oxidation, changes in the protein levels of substrate oxidation machinery, includine glutamine transporters (39) or catabolic enzymes (40), may underlie differences in capacity that are not detectable in the substrate-driven rates, in which control by ATP demand is much greater.

The maximum J_ATPglyc_ (J_ATPglyc|max_) calculated for glutamine and pyruvate (Figure 5) were surprising, since glycolytic capacity from exclusive catabolism of non-glycolytic substrates was expected to be zero. These calculated rates likely result either from an overestimate of acidification rate, or from a true acidification rate not accounted for by respiratory CO_2_, In either case, these rates probably do not represent true J_ATPglyc_. Overestimated _JATPglyc|max_ would also cause overestimation of SFI values and bioenergetic capacities, which are both determined using J_ATPglyc|max_. Since our major findings of (1) full ATP supply flexibility at substrate-driven J_ATPproduction_ for both MCF7 and C2C12 cells, (2) J_ATPproduction_ responsive to substrate composition in MCF7, and (3) the ability of MCF7 to access additional oxidative capacity using glutamine, are not reliant on J_ATPglycmax_, potential error in the calculation of J_ATPglycmax_ do not change our major conclusions.

However, these discrepancies do highlight some of the limitations of the ATP calculation model. The assumptions underlying the conversion of extracellular H^+^ and O_2_ flux to J_ATPglyc_ and J_ATPox_, allowing determination of J_ATPproduction_, are fully validated for glucose, but not for glutamine or pyruvate, in an intact-cell system. We find negligible contributions attributable to fluxes other than through glycolysis and oxidative phosphorylation under the conditions assayed (with the exception of J_ATPglycmax_), suggesting that deviations from the model are small. However, this calculation is currently reliable only for non-growing cells, as growth would change the H^+^/O_2_ and P/O ratios on which the calculation relies. This represents a potential limitation on applying these findings to rapidly growing cells. In addition, this approach would fail to detect the potentially significant operation of pathways whose sum of errors happens to be small.

In conclusion, we demonstrate here that despite the common constraints of cell culturing in the same medium, MCF7 cancer cells display notable differences from a non-cancer cell type that are consistent with, and further refine, existing models of metabolic flexibility in cancer, including a potential bioenergetic basis for the hypothesis that glutamine oxidation provides access to bioenergetic capacity not afforded by other oxidized substrates. In contrast to many prevailing models of cancer cell metabolism, these differences do not always include substrate-driven ATP supply flexibility, greater ATP demand, or a greater reliance on glycolysis to meet ATP demand.

## Data Availability Statement

The datasets generated for this study are available on request to the corresponding author.

## Author Contributions

ML co-wrote the manuscript. JT, MB, JM, AG, CL, and DL collected and analyzed data presented and edited the manuscript. SM conceived the project, designed the experiments, and wrote the manuscript. All authors contributed to the article and approved the submitted version.

## Conflict of Interest

AG declares financial interest in Image Analyst Software. The remaining authors declare that the research was conducted in the absence of any commercial or financial relationships that could be construed as a potential conflict of interest.
